# Supplementary material for: Frasier Syndrome: A 15-Year-Old Phenotypically Female Adolescent Presenting with Delayed Puberty and Nephropathy
Source: Children (Basel). 2023 Mar 17;10(3):577. doi: 10.3390/children10030577 (PMC10046944; doi:10.3390/children10030577)
Supplement: Supplementary file 1 [file children-10-00577-s001.zip › children-2228125-supplementary.pdf]

Supplementary Table 1. Clinical characteristics of FS patients with the IVS9 +4C>T mutation in the WT1 gene

Chromosome karyotype	External genitalia	Age of diagnosis	Initial symptom (age)	Renal biopsy	ESRD	delayed secondary sex characteristics*	Urological/genital tumors	Reference
46,XY	F	15y	Delayed puberty(12y)	FSGS	16y	Delayed puberty(12y)	-	#
46,XY	F	14m	Proteinuria(17w)	FSGS	-	-	-	[1]
46XX	F	NA	SNRS(12y)	MPGN	13y	-	-	[1]
46,XY	F	8y	Proteinuria(3y)	FSGS	-	-	gonadoblastoma	[2]
46,XY	F	16y	Proteinuria(NA)	FSGS	-	Primary amenorrhea(16y）	-	[3]
46,XY	M	29y	HBP(29y)	NA	29y		Sertoli cell tumor Seminoma	[4]
46,XY	M	6.3y	Edema(6.3y)	-	6.3y	-	-	[5]
46,XY	F	5y	Proteinuria(2y)	FSGS	-	-	gonadoblastoma bilaterally	[6]
46,XY	F	13y	peritoneal syndrome(13y)	FSGS	14y	-	ovarian dysgerminoma	[7]
46,XY	F	17.3y	CKD(NA)	NA	11y	Primary amenorrhea (17.3)	gonadoblastoma	[8]
46,XY	F	18y	CKD(NA)	NA	18y	Primary amenorrhea (15y)	-	[8]
46,XY	F	3y	proteinuria	FSGS	3y	-	-	[9]
46,XY	F	15y	Proteinuria(11y)	NA	11y	Delayed puberty(15y)	germinoma	[10]
46,XY	F	19y	Proteinuria(3y）	MPGN	-	Delayed puberty(12y）	-	[11]
46,XY	F	19y	Proteinuria(3y)	MPGN	20y	Delayed puberty（12y）	-	[11]
46,XY	M	22y	Proteinuria(13y)	NA	18y	-	Gonadoblastoma	[12]
46,XY	F	13y	NS(9y)	FSGS	17y	Delayed puberty（13y）	-	[13]
46,XY	F	11Y	SNRS(22m)	FSGS	8y	-	-	[14]
46,XY	F	17Y	Proteinuria(10y）	FSGS	17y	-	Gonadoblastoma	[15]
46,XY	F	18y	Proteinuria(4y）	FSGS	-	Primary amenorrhea (16y)	-	[16]
46,XY	F	16y	Proteinuria(4y）	FSGS	-	-	-	[16]
46,XY	F	12y	Proteinuria(6y)	NA	-	Delayed puberty(12y)	Wilms' tumor	[17]

46,XY	F	17y	proteinuria(7m)	FSGS	12y	Delayed puberty(17y)	dysgerminom	[17]
46,XY	F	18y	Proteinuria(3y)	FSGS	-	-	-	[18]
46,XY	F	18y	Proteinuria(3y)	FSGS	-	-	-	[18]
46,XY	F	19	Proteinuria(5y)	FSGS	16y	-	Gonadoblastoma	[18]
46,XX	F	1y	Proteinuria (4m)	DMS	1y	-	-	[19]
46,XY	F	NA	Proteinuria (6y)	FSGS	9y	-	myofibroblastic tumor	[20]
46,XY	F	NA	Proteinuria (2y)	unspecific glomerular changes	23y	-	Gonadoblastoma	[20]

* delayed secondary sex characteristics: primary amenorrhea and/or delayed puberty; # the patient in this case
F: female; M: male; NA: not available; MPGN: mesangial proliferative glomerulonephritis; DMS: diffuse mesangial sclerosis.

References

1. Anderson, E.; Aldridge, M.; Turner, R.; Harraway, J.; McManus, S.; Stewart, A.; Borzi, P.; Trnka, P.; Burke, J.; Coman, D. WT1 complete gonadal dysgenesis with membranoproliferative glomerulonephritis: case series and literature review. *Pediatric Nephrology (Berlin, Germany)* **2022**, *37*, 2369-2374, doi:10.1007/s00467-022-05421-8.
2. Matsuoka, D.; Noda, S.; Kamiya, M.; Hidaka, Y.; Shimojo, H.; Yamada, Y.; Miyamoto, T.; Nozu, K.; Iijima, K.; Tsukaguchi, H. Immune-complex glomerulonephritis with a membranoproliferative pattern in Frasier syndrome: a case report and review of the literature. *BMC Nephrology* **2020**, *21*, 362, doi:10.1186/s12882-020-02007-0.
3. Merhi, Z.; Pollack, S.E. Pituitary origin of persistently elevated human chorionic gonadotropin in a patient with gonadal failure. *Fertility and Sterility* **2013**, *99*, 293-296, doi:10.1016/j.fertnstert.2012.08.051.
4. Kitsiou-Tzeli, S.; Deligiorgi, M.; Malaktari-Skarantavou, S.; Vlachopoulos, C.; Megremis, S.; Fylaktou, I.; Traeger-Synodinos, J.; Kanaka-Gantenbein, C.; Stefanadis, C.; Kanavakis, E. Sertoli cell tumor and gonadoblastoma in an untreated 29-year-old 46,XY phenotypic male with Frasier syndrome carrying a WT1 IVS9+4C>T mutation. *Hormones (Athens)* **2012**, *11*, 361-367.
5. Yang, Y.; Feng, D.; Huang, J.; Nie, X.; Yu, Z. A child with isolated nephrotic syndrome and WT1 mutation presenting as a 46, XY phenotypic male. *Eur J Pediatr* **2013**, *172*, 127-129, doi:10.1007/s00431-012-1770-0.
6. Sinha, A.; Sharma, S.; Gulati, A.; Sharma, A.; Agarwala, S.; Hari, P.; Bagga, A. Frasier syndrome: early gonadoblastoma and cyclosporine responsiveness. *Pediatric Nephrology (Berlin, Germany)* **2010**, *25*, 2171-2174, doi:10.1007/s00467-010-1518-x.

7. Mestrallet, G.; Bertholet-Thomas, A.; Ranchin, B.; Bouvier, R.; Frappaz, D.; Cochat, P. Recurrence of a dysgerminoma in Frasier syndrome. *Pediatr Transplant* **2011**, *15*, e53-e55, doi:10.1111/j.1399-3046.2010.01273.x.
8. Andrade, J.G.R.d.; Guaragna, M.S.; Soardi, F.C.; Guerra-Júnior, G.; Mello, M.P.d.; Maciel-Guerra, A.T. Clinical and genetic findings of five patients with WT1-related disorders. *Arq Bras Endocrinol Metabol* **2008**, *52*, 1236-1243.
9. Aucella, F.; Bisceglia, L.; De Bonis, P.; Gigante, M.; Caridi, G.; Barbano, G.; Mattioli, G.; Perfumo, F.; Gesualdo, L.; Ghiggeri, G.M. WT1 mutations in nephrotic syndrome revisited. High prevalence in young girls, associations and renal phenotypes. *Pediatric Nephrology (Berlin, Germany)* **2006**, *21*, 1393-1398.
10. Chan, W.K.Y.; To, K.F.; But, W.M.; Lee, K.W. Frasier syndrome: a rare cause of delayed puberty. *Hong Kong Med J* **2006**, *12*, 225-227.
11. Ito, S.-i.; Hataya, H.; Ikeda, M.; Takata, A.; Kikuchi, H.; Hata, J.-i.; Morikawa, Y.; Kawamura, S.; Honda, M. Alport syndrome-like basement membrane changes in Frasier syndrome: an electron microscopy study. *Am J Kidney Dis* **2003**, *41*, 1110-1115.
12. Melo, K.F.S.; Martin, R.M.; Costa, E.M.F.; Carvalho, F.M.; Jorge, A.A.; Arnhold, I.J.P.; Mendonca, B.B. An unusual phenotype of Frasier syndrome due to IVS9 +4C>T mutation in the WT1 gene: predominantly male ambiguous genitalia and absence of gonadal dysgenesis. *The Journal of Clinical Endocrinology and Metabolism* **2002**, *87*, 2500-2505.
13. Bönte, A.; Schröder, W.; Denamur, E.; Querfeld, U. Absent pubertal development in a child with chronic renal failure: the case of Frasier syndrome. *Nephrol Dial Transplant* **2000**, *15*, 1688-1690.
14. Denamur, E.; Bocquet, N.; Baudouin, V.; Da Silva, F.; Veitia, R.; Peuchmaur, M.; Elion, J.; Gubler, M.C.; Fellous, M.; Niaudet, P.; et al. WT1 splice-site mutations are rarely associated with primary steroid-resistant focal and segmental glomerulosclerosis. *Kidney International* **2000**, *57*, 1868-1872.
15. Okuhara, K.; Tajima, S.; Nakae, J.; Sasaki, S.; Tochimaru, H.; Abe, S.; Fujieda, K. A Japanese case with Frasier syndrome caused by the splice junction mutation of WT1 gene. *Endocr J* **1999**, *46*, 639-642.
16. Demmer, L.; Primack, W.; Loik, V.; Brown, R.; Therville, N.; McElreavey, K. Frasier syndrome: a cause of focal segmental glomerulosclerosis in a 46,XX female. *J Am Soc Nephrol* **1999**, *10*, 2215-2218.
17. Barbosa, A.S.; Hadjiathanasiou, C.G.; Theodoridis, C.; Papathanasiou, A.; Tar, A.; Merksz, M.; Györvári, B.; Sultan, C.; Dumas, R.; Jaubert, F.; et al. The same mutation affecting the splicing of WT1 gene is present on Frasier syndrome patients with or without Wilms' tumor. *Hum Mutat* **1999**, *13*, 146-153.
18. Kikuchi, H.; Takata, A.; Akasaka, Y.; Fukuzawa, R.; Yoneyama, H.; Kurosawa, Y.; Honda, M.; Kamiyama, Y.; Hata, J. Do intronic mutations affecting splicing of WT1 exon 9 cause Frasier syndrome? *J Med Genet* **1998**, *35*, 45-48.
19. Jeanpierre, C.; Denamur, E.; Henry, I.; Cabanis, M.O.; Luce, S.; Cécille, A.; Elion, J.; Peuchmaur, M.; Loirat, C.; Niaudet, P.; et al. Identification of

constitutional WT1 mutations, in patients with isolated diffuse mesangial sclerosis, and analysis of genotype/phenotype correlations by use of a computerized mutation database. *Am J Hum Genet* **1998**, *62*, 824-833.

20. Barbaux, S.; Niaudet, P.; Gubler, M.C.; Grünfeld, J.P.; Jaubert, F.; Kuttenn, F.; Fékété, C.N.; Souleyreau-Therville, N.; Thibaud, E.; Fellous, M.; et al. Donor splice-site mutations in WT1 are responsible for Frasier syndrome. *Nat Genet* **1997**, *17*, 467-470.
